# Statins inhibit proliferation and induce apoptosis in triple-negative breast cancer cells

**DOI:** 10.1007/s12032-022-01733-9

**Published:** 2022-07-14

**Authors:** Shane O’Grady, John Crown, Michael J. Duffy

**Affiliations:** 1 UCD School of Medicine, Conway Institute of Biomolecular and Biomedical Research, University College Dublin, Dublin, Ireland; 2 Department of Medical Oncology, St Vincent’s University Hospital, Dublin, Ireland; 3 Clinical Research Centre, St Vincent’s University Hospital, Elm Park, Dublin, D04 T6F4 Ireland

**Keywords:** Statins, Breast cancer, Triple-negative, p53, Treatment

## Abstract

*TP53* (p53) is mutated in 80–90% of cases of triple-negative breast cancer (TNBC). Statins, which are widely used to treat elevated cholesterol, have recently been shown to degrade mutant p53 protein and exhibit anti-cancer activity. The aim of this work was to evaluate the potential of statins in the treatment of TNBC. The anti-proliferative effects of 2 widely used statins were investigated on a panel of 15 cell lines representing the different molecular subtypes of breast cancer. Significantly lower IC50 values were found in triple-negative (TN) than in non-TN cell lines (atorvastatin, *p* < 0.01; simvastatin *p* < 0.05) indicating greater sensitivity. Furthermore, cell lines containing mutant p53 were more responsive to both statins than cell lines expressing wild-type p53, suggesting that the mutational status of p53 is a potential predictive biomarker for statin response. In addition to inhibiting proliferation, simvastatin was also found to promote cell cycle arrest and induce apoptosis. Using an apoptosis array capable of detecting 43 apoptosis-associated proteins, a novel protein shown to be upregulated by simvastatin was the IGF-signalling modulator, IGBP4, a finding we confirmed by Western blotting. Finally, we found synergistic growth inhibition between simvastatin and the IGF-1R inhibitor, OSI-906 as well as between simvastatin and doxorubicin or docetaxel. Our work suggests repurposing of statins for clinical trials in patients with TNBC. Based on our findings, we suggest that these trials investigate statins in combination with either doxorubicin or docetaxel and include p53 mutational status as a potential predictive biomarker.

## Introduction

Patients with triple-negative breast cancer (TNBC) lack estrogen receptors, progesterone receptors as well as HER2 gene amplification/overexpression. Consequently, these patients cannot be treated with 2 of the most effective therapies currently available for breast cancer, i.e. endocrine and anti-HER2 therapy. Indeed, until recently, the only form of systemic therapy available for patients with TNBC was cytotoxic chemotherapy [1]. Recently, however, several non-cytotoxic treatments have been approved for TNBC including the monoclonal antibody-conjugate, sacituzumab govitecan; immunotherapy with immune checkpoint such as atezolizumab or pembrolizumab and PARP inhibitors (olaparib, talazoparib) for the approximate 10–20% of TNBC patients with germline BRCA1/2 mutations [2, 3]. Although these therapies are improving outcome in some patients with TNBC, all are efficacious in only a proportion of patients with the disease. Clearly, therefore, additional forms of treatment are necessary for patients with TNBC [2, 3].

One of the most successful forms of anti-cancer treatment introduced in recent years is the use of drugs for targeting cancer driver genes. The most frequently occurring driver gene in TNBC is mutations in p53 which are present in 80–90% of these patients [4, 5]. Thus, mutant p53 is a highly attractive target for new anti-cancer drugs for the treatment of patients with TNBC.

Historically, however, mutant p53 has proved difficult to target and was thus frequently regarded as “undruggable” [6, 7]. This traditional viewpoint, however, is changing as several new strategies have recently been identified for targeting the mutant protein [8, 9]. Two of the most promising strategies include degradation of the mutant protein and reactivation of mutant protein back to its wild-type form (for reviews, see refs. [8, 9]).

While mutant p53-reactivating compounds have been widely investigated [8], less work has been devoted to compounds that promote degradation of the mutant protein. For mutant p53 to exert its oncogenic activity, it must be stabilized [10]. Stabilization is achieved by interaction with heat shock proteins (HSP), especially HSP40, HSP70 and HSP90 [11]. Preventing these interactions might be expected to result in mutant p53 degradation and thus suppression of cancer growth. Early evidence that destabilization of mutant p53 had anti-cancer activity was obtained with HSP inhibitors in a range of experimental models [12]. However, in clinical trials, HSP inhibitors were found to be relatively toxic and lack efficacy [13, 14]. Consequently, these trials were largely abandoned.

In addition to HSP inhibitors, a group of commonly used drugs known as statins, have also been shown to degrade mutant p53 but not WT p53 [15, 16]. Statins which are widely used to treat patients with high levels of cholesterol act by inhibiting HMG-CoA, the rate-limiting enzyme in biosynthesis of the lipid [16]. In addition to reducing cholesterol levels, treatment with statins also lowers levels of other intermediate metabolites in the cholesterol biosynthetic pathway, especially mevalonate phosphate (MVP). Recently, Parrales et al. [15] reported that decreased MVP levels resulting from treatment with the statin, lovastatin led to reduced binding of mutant p53 to a specific form of HSP40 known as DNAJA1. As a result of the decreased binding to DNAJAI, mutant p53 underwent degradation with the ubiquitin ligase CHIP. Consistent with its ability to degrade mutant p53, lovastatin was found to inhibit the in vitro and in vivo growth of tumour cells expressing mutant p53 (conformational mutations) but not cells with wild-type p53, cell lines null for p53 or cell lines containing p53 contact mutations [15]. In a more recent study, treatment with a different statin, i.e. cerivastatin resulted in the dissociation of mutant p53 from HSP90 and degradation by MDM2 [16].

Based on the high prevalence of p53 mutations in TNBC, the ability of statins to degrade mutant p53, and thereby inhibit tumour cell proliferation, the aim of our study was to test the hypothesis that these drugs might provide a new treatment for patients with TNBC.

## Materials and methods

### Cell culture

All cell lines were originally sourced from the American Type Culture Collection (ATCC), apart from Hs578Ts(i8) which was supplied by Dr. Susan McDonnell, University College, Dublin. All cell lines were maintained in RPMI, supplemented with 10% fetal bovine serum and 1% penicillin–streptomycin. All cell culture reagents were purchased from Biosciences (Dun Laoghaire, Ireland) and Sigma-Aldrich (Arklow, Ireland). Cells were maintained at 37 °C in a 5% CO2 environment. The p53 mutation status of these cell lines is shown in Table 1.

**Table 1:** p53 mutational status of cell lines used in this study

Cell line	p53 status	Mutation
MCF-7	WT	–
T47D	MUT	L194F
ZR-75-1	WT	–
CAMA1	MUT	R280T
SKBR3	MUT	R175H
JIMT-1	MUT	R248W
Hs578T	MUT	V157F
Hs578Ts(i)8	MUT	V157F
MDA-MB-231	MUT	R280K
MDA-MB-453	MUT	H368delinsG
MDA-MB-468	MUT	R273H
HCC1143	MUT	R248Q
BT-549	MUT	R249S
SUM159	MUT	R273H
CAL-85-1	MUT	L132E

### Proliferation assays

Cell response to statins was assessed by seeding 96-well plates with 5,000 cells per well and allowing to adhere overnight. The following day, fresh media were added, containing statins at the indicated concentration or DMSO. Cells were incubated for 5 days, at which point viability was assessed by adding 3-(4,5-dimethylthiazol-2-yl)-2,5-diphenyltetrazolium bromide (MTT) (Sigma-Aldrich) to 10% of media volume and incubating for 2 h. Absorbance was measured at 570 nm. In some experiments, mevalonate was added at a concentration of 100 or 200 µM.

### Cell cycle analysis

Cells treated with statins for 24 h were harvested and fixed by resuspending in 500 µL PBS followed by slowly adding 4.5 mL ice-cold 70% ethanol. Cells were prepared for cell cycle analysis using FxCycle™ PI/RNase Staining Solution (Thermo-Fisher), following manufacturer’s protocol. Fluorescence was measured on a BD FACSCanto II, using a 488 nm excitation source and a 576 nm emission filter. Cell cycle percentages were calculated using Flowjo software.

### Apoptosis detection

Cells treated with statins for 48 h were harvested and prepared for analysis using the FITC Annexin V Apoptosis Detection Kit with 7-AAD (Medical Supply Company, Mulhuddart, Ireland) following manufacturer’s guidelines. Fluorescence intensity of FITC and 7-AAD was measured on a BD FACSCanto II, using a 488 nm excitation source and a 525 nm or 695 nm emission filter, respectively.

### Membrane array for detection of apoptosis-associated proteins

Expression of apoptotic proteins was analysed using the Raybiotech Human Apoptosis Array following manufacturer’s guidelines. Briefly, protein was isolated from SUM159 cells following treatment for 24 h with 10 µM simvastatin or vehicle control, using the provided lysis buffer. Membranes were blocked and incubated with protein samples overnight at 4 °C. After washing with the provided buffers, membranes were incubated overnight again at 4 °C with detection antibody. Membranes were incubated at room temperature with HRP-Streptavidin for 2 h. Following a final wash stage, membranes were incubated with detection buffer and imaged using a Licor C-DiGit Blot Scanner. ImageJ software was used to generate densitometry values which were then normalised to the internal controls on the membrane.

### Western blotting

Protein was isolated and prepared for analysis by Western blotting as previously described [17]. Primary incubations were carried out overnight at 4 °C with the following antibodies; IGFBP4 (R&D Systems, Abingdon, UK. 1:1000), GAPDH (Sigma, 1:4000). Secondary incubation was performed for 1 h at room temperature using HRP-conjugated IgG kappa binding protein (Santa-Cruz Biotech, Heidelberg, Germany. 1:5000). Blots were imaged using SuperSignal West Pico PLUS substrate (Biosciences) and a Licor C-DiGit Blot Scanner.

### Drug combination assays

Efficacy of drug combinations was performed following the same protocol as proliferation assays. Drugs were tested as both single agents and in combination. Combination index (CI) values were calculated using Compusyn software [18]. CI values below 1 are indicative of drug synergy.

### Statistics

All experiments were repeated at least 3 times. All statistical analysis was performed using GraphPad Prism 8 software. Significance was determined using Student t tests or ANOVA post-hoc analysis. A p value of less than 0.05 was deemed statistically significant.

## Results

### Statins reduce breast cancer cell line proliferation

**Fig. 1:**
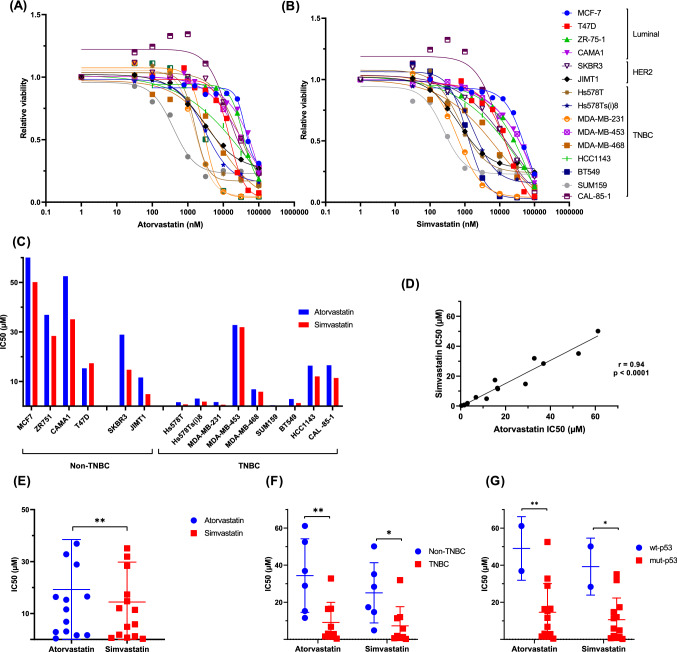
Anti-proliferative effect of statins in breast cancer. Viability of a panel of breast cancer cell lines was assessed by MTT assay following 5 days incubation with either atorvastatin (**A**) or simvastatin (**B**). Dose–response curves were used to calculate IC50 values (**C**). Cell sensitivity to the two statins was found to be correlated (**D**) although significantly greater for simvastatin (**E**). TNBC cell lines were found to be more sensitive to statin treatment than non-TNBC cell lines (**F**). Presence of mutant p53 allele was also found to influence statin response (**G**). All figures represent mean of 4 independent experiments. *p < 0.05, **p < 0.01

We initially tested the effect of 2 widely used statins on the viability of a panel of 15 breast cancer cell lines, representative of the major breast cancer molecular subtypes; luminal (4 cell lines), HER2 (2 cell lines) and TNBC (9 cell lines). Both statins reduced cell viability in a dose-dependent and cell type-dependent manner (Fig. 1A and B). The IC50 values for both statins were highly variable across the panel, ranging from 0.4 to 61 µM for atorvastatin, and from 0.2 to 50 µM for simvastatin (Fig. 1C). Although the IC50 values for the 2 statins were highly correlated across the panel (*r* = 0.94, *p* < 0.0001) (Fig. 1D), significantly lower values were found with simvastatin compared with atorvastatin (*p* < 0.01) (Fig. 1E). Of potential clinical significance, we found significantly lower IC50 values for both statins in TNBC versus non-TNBC cell lines (atorvastatin, *p* < 0.01; simvastatin *p* < 0.05) (Fig. 1F). The enhanced efficacy of statins in TNBC cell lines may be due to the high prevalence of p53 mutations in this subtype [19], Indeed, we found lower IC50 values for both atorvastatin and simvastatin in cell lines harbouring mutant versus WT-p53 alleles (Fig. 1G). As mentioned previously, statins have been shown to exert some of their anti-tumour effect through degradation of mutant p53 [15, 20]. Although a previous study found that only cell lines harbouring conformational p53 mutations were responsive to statins [15], we did not observe any difference in IC50 values between cell lines with conformational versus contact-type mutations (data not shown).

### Anti-proliferative effects of statins are abrogated by mevalonate

**Fig. 2:**
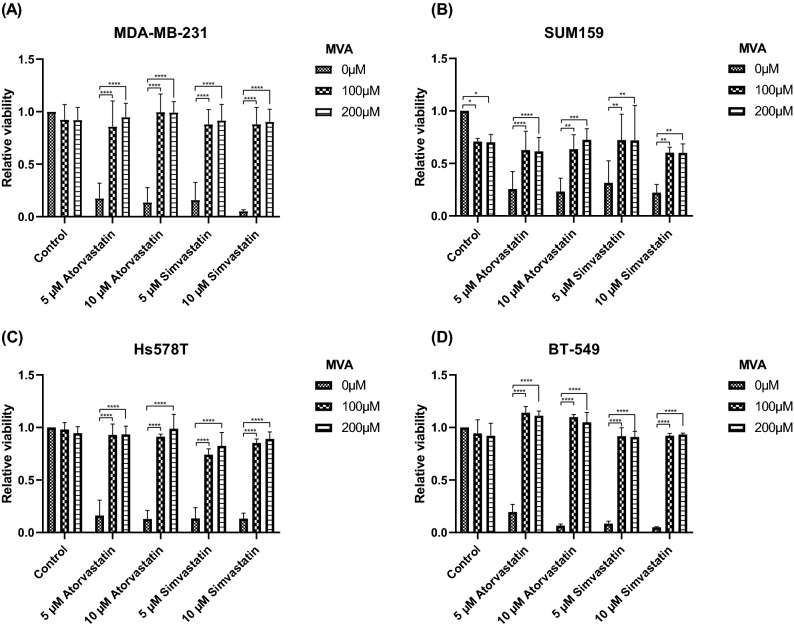
Exogenous MVA reverses anti-proliferative effect of statins. Viability of TNBC cell lines cultured for 5 days in indicated concentrations of atorvastatin/simvastatin and MVA, as assessed by MTT assay. Exogenous MVA significantly improved viability levels in all tested cell lines. All figures represent mean of 4 independent experiments. *p < 0.05, **p < 0.01, ***p < 0.001, ****p < 0.0001

Although statins decrease cholesterol production by inhibiting the rate-limiting enzyme in its biosynthesis (HMGCR), it also reduces levels of the intermediate product, MVA [21]. To confirm that the anti-proliferative effect was via MVA reduction and not an unexpected off-target effect, we selected 4 TNBC cell lines previously observed to be statin-sensitive and assessed the effect of exogenous MVA on statin-induced growth inhibition. Addition of MVA by itself had no effect on viability except in the SUM159 cell line, where an approximately 30% decrease was observed for both 100 and 200 µM (Fig. 2B). In all tested cell lines, both atorvastatin and simvastatin were again found to effectively reduce viability at either 5 or 10 µM (Fig. 2). This reduction was reversed by the addition of either 100 or 200 µM MVA, which completely restored normal levels of cell viability in 3 cell lines (MDA-MB-231, Hs578T and BT-549, Fig. 2A, C, D, respectively) and lead to partial restoration in the SUM159 cell line (Fig. 2B). Although it was not clear why proliferation remained suppressed in the SUM159 cell line, we previously found this cell line to be highly statin-sensitive (Fig. 1A, B), which may explain the limited restoration of viability at MVA dosage used. Abrogation of the anti-proliferative effect of statins by MVA further illustrates the importance of this pathway in breast cancer cell viability.

### Effect of simvastatin on cell cycle arrest

**Fig. 3:**
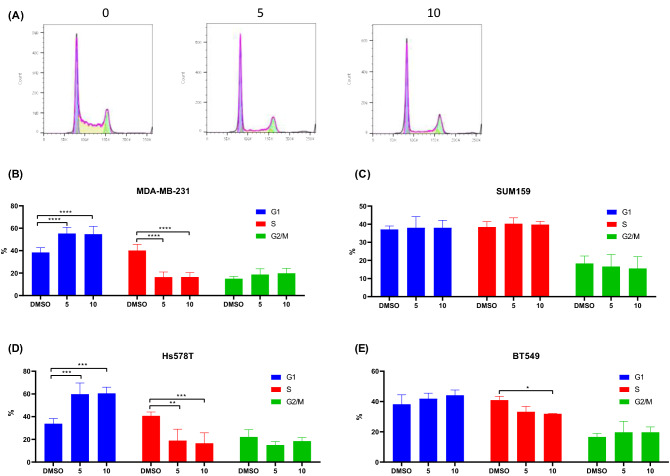
Statins induce cell cycle arrest in TNBC cell lines. DNA content of TNBC cell lines following 24 h incubation with 5 or 10 µM simvastatin. Representative graphs of MDA-MB-231 cells (**A**). Simvastatin significantly increased G1-arrest and decreased S-phase in 3 of 4 tested cell lines (**B**, **D**, **E**) but not in one cell line (**C**). All figures represent mean of 3 independent experiments. **p < 0.01, ***p < 0.001, ****p < 0.0001

As simvastatin was the more potent of the two statins investigated, we focussed on it for the rest of our study. To investigate if simvastatin could induce cell cycle arrest in TNBC cells, we measured DNA content by propidium iodide staining and flow-cytometry in 4 TNBC cell lines following 24 h treatment. Representative graphs of simvastatin-treated MDA-MB-231 cells are shown below (Fig. 3A). Simvastatin induced significant decreases in S-phase in 3 of 4 tested cell lines (Fig. 3B, D, E). In contrast, no significant effect was observed in SUM159 cells (Fig. 3C). It is unclear why this one cell line did not undergo cell cycle arrest. A possible reason is that we limited our analysis to 24 h post-treatment and a longer time-point may have yielded different results.

### Apoptotic effect of simvastatin in TNBC

**Fig. 4:**
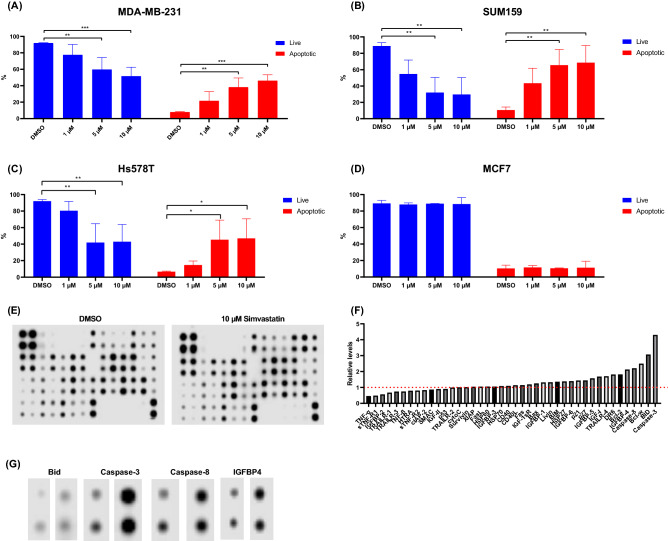
Simvastatin is a potent inducer of apoptosis in TNBC cells. Apoptosis was measured in MDA-MB-231 (**A**), SUM159 (**B**), Hs578T (**C**) and MCF7 (**D**) cells following 48 h incubation with the indicated concentration of simvastatin (*n* = 3). Apoptotic cells were determined by positive staining with annexin V-FITC. Expression of apoptotic markers was assessed with a RayBiotech antibody array in SUM159 cells following 24 h treatment with 10 µM simvastatin (**E**). Normalised densitometry values (**F**) revealed upregulation of several pro-apoptotic markers. Select highlights from apoptotic array (**G**). *p < 0.05, **p < 0.01, ***p < 0.001

**Fig. 5:**
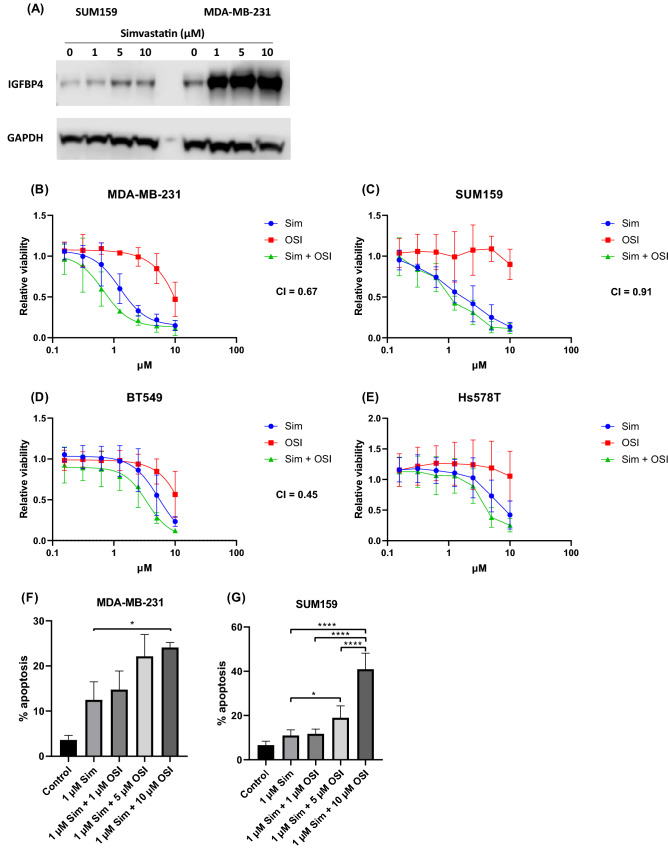
Synergistic effect of statins and IGF inhibition in TNBC. Representative IGFBP4 western blot image of SUM159 and MDA-MB-231 cells treated for 24 h with increasing doses of simvastatin (**A**). MTT proliferation assays of MDA-MB-231 (**B**), SUM159 (**C**), BT549 (**D**) and Hs578T (**E**) cell lines following 5 days treatment with simvastatin, OSI-906 or a combination therapy. Levels of apoptosis were measured in MDA-MB-231 (**F**) and SUM159 (**G**) cells following 48 h treatment with combined 1 µM simvastatin and escalating concentrations of OSI-906. All figures represent mean of three independent experiments. *p < 0.05, ****p < 0.0001

We next investigated if the loss of viability seen in statin-treated breast cancer cells was due to activation of apoptotic processes. We treated 3 TNBC and 1 non-TNBC cell lines with escalating doses of simvastatin for 48 h and measured apoptosis by flow-cytometry. All 3 TNBC cell lines had significantly increased levels of annexin-V staining at both 5 and 10 µM simvastatin. In MDA-MB-231 (Fig. 4A), 5 and 10 µM simvastatin led to 38% (*p* < 0.01) and 46% (*p* < 0.001) cells staining positive for annexin-V, respectively. Similar levels of annexin-V positivity were seen in Hs578T (Fig. 4C) cells. SUM159 cells were particularly sensitive to apoptosis, with 65% and 68% (*p* < 0.01) annexin-V staining (Fig. 4B). In all cases, the increase in apoptotic cells was accompanied by a statistically significant decrease in live cells. In contrast to our findings with TNBC, mutant-p53 cell lines, there was no induction of apoptosis in the luminal cell line MCF-7 at any tested concentrations (Fig. 4D). This inability to induce apoptosis in MCF-7 cells may be related to its WT-p53 status.

To further study the mechanism of apoptosis in statin-treated TNBC cells, we incubated SUM159 cells with 10 µM simvastatin for 24 h and analysed expression of a panel of apoptotic markers using a commercially available antibody array. Several key apoptotic proteins including caspase-3, caspase-8 and Bid were found to be increased following 24 treatments with simvastatin (Fig. 4E, F, G). The highest level of upregulation was seen in the apoptosis executioner caspase-3 (fourfold), followed by the pro-apoptotic Bcl-2 family member, Bid (threefold). A novel finding was the upregulation of IGBF4. Enlarged images of highlighted proteins are shown (Fig. 4G).

### Statins synergise with IGF pathway inhibition

As IGBF4 was not previously reported to be regulated by a statin, we validated this finding via Western blots. As shown in Fig. 5A, both SUM159 and MDA-MB-231 cells had significantly upregulated IGFBP4 in response to 24 h simvastatin treatment. Since IGFBP4 plays a role in regulating the IGF pathway, we assessed if inhibition of IGF signalling could synergise with statins by comparing viability of 4 TNBC cell lines treated with statins alone or in combination with the IGF1-R inhibitor, OSI-906. In agreement with prior studies [22, 23], OSI-906 as a single agent had limited effects on TNBC cell proliferation (Fig. 5B, C, D, E). However, the addition of OSI-906 enhanced its anti-proliferative effect in a synergistic manner i.e. CI values for MDA-MB-231, SUM159 and BT549 cell lines (Fig. 5B, C, D) were 0.67, 0.91 and 0.45, respectively. A CI value could not be calculated for Hs578T cells (Fig. 5E), as no tested concentration of OSI-906 had an observable effect on proliferation in this cell line.

Finally, we tested the combination of simvastatin and OSI-906 on apoptosis. MDA-MB-231 and SUM159 cells were treated with a low dose of simvastatin (1 µM) that, by itself, did not significantly induce apoptosis in either cell line (Fig. 4A, B). Similar to results observed in our proliferation assays, OSI-906 as a single agent had no significant effect on the rate of apoptosis (data not shown) but markedly enhanced the apoptotic effect of simvastatin in both cell lines. In MDA-MB-231 cells, combination of 10 µM OSI-906 and 1 µM simvastatin lead to a twofold increase in apoptotic cells versus 1 µM simvastatin alone (Fig. 5F, *p* < 0.05). The combination was also effective in SUM159 cells (Fig. 5G), where the addition of either 5 µM or 10 µM OSI-906 increased apoptosis relative to simvastatin alone (1.7-fold, *p* < 0.05; 3.7-fold, *p* < 0.0001, respectively).

### Statins synergise with chemotherapy drugs

As statins are unlikely to be used alone in cancer treatment, we also tested the anti-proliferative effect of combined treatment with a statin and standard chemotherapy drugs used for the treatment of breast cancer. As shown in Table 2, synergistic growth inhibition was observed when simvastatin was combined with either doxorubicin or docetaxel in MDA-MB-231 and SUM159 cells.

**Table 2:** CI values of cell lines treated with combination of simvastatin and doxorubicin or docetaxel for 5 days (*n* = 3)

	Doxorubicin	Docetaxel
MDA-MB-231	0.43924	0.33896
SUM159	0.60257	0.6241

## Discussion

Here, we show that although 2 widely used statins decreased the proliferation of breast cancer cell lines representing the main molecular subtypes of breast cancer, their inhibitory impact was significantly more potent in TNBC than in non-TNBC cell lines. A possible explanation for the enhanced sensitivity of the TNBC versus the non-TNBC cell lines may relate to the higher prevalence of p53 mutations in this breast cancer subtype. Indeed, as mentioned in the Introduction above, our findings are consistent with previous observations in which treatment with statins was reported to result in the degradation of mutant but not wild-type p53 [15, 16]. Previously, Chou et al. found that lung cancer cell lines containing mutant p53 were also more sensitive to statins than cells with wild-type p53 [20]. The effects of the statins on reduced cell proliferation in our study appeared to be mediated by induction of cell cycle arrest and promotion of apoptosis.

Previously, Parrales et al. reported that lovastatin degraded p53 with conformational mutations but had minimal effects on contact mutations [15]. Based on this finding, it might be expected that cell lines expressing conformational mutations would be more sensitive to growth inhibition than cell lines expressing mutant p53 with contact mutations. However, our work using a larger panel of cell lines found similar IC50 values, irrespective of the type of p53 mutation.

Although our results show that two different statins reduced the proliferation of TNBC cell lines, these drugs are unlikely to be used alone for the treatment of this form of breast cancer. We therefore evaluated the effects of combining simvastatin with two frequently used drugs to treat TNBC, i.e. docetaxel and doxorubicin. Both these cytotoxic drugs in combination with simvastatin synergistically enhanced growth inhibition in the 2 TNBC cell lines investigated, suggesting that combined treatment with simvastatin and docetaxel or doxorubicin might be investigated in a clinical trial.

A novel finding in this report was that simvastatin upregulated levels of IGBPF4. Although we did not investigate the mechanism of this upregulation or its possible implication for statin actions, previous studies showed that IGFBP4 binds and sequesters IGF-1 ligands, thus acting to dampen IGFR1 signalling [24]. Consistent with this observation, administration of a high dose of atorvastatin decreased serum IGF-1 levels in diabetic patients [25, 26]. Furthermore, statins were reported to downregulate expression of the IGF1R [27, 28]. Theoretically, therefore, statin-induced upregulation of IGBP4 could lead to sequestration of IGF1 which in turn could downregulate IGF1R signalling. Decreased IGF1R signalling might be expected to result in inhibition of cell line growth and/or promotion of apoptosis as this signalling system has been has been shown to promote breast cancer cell proliferation and survival [29]. Modulation of IGFBP4 expression has previously been suggested as a therapeutic strategy, with a degradation-resistant form of the protein demonstrating significant effects on tumour growth and angiogenesis in both in vitro and in vivo models [30, 31].

We should state however, that there are several other mechanisms other than via upregulation of IGBP4 by which statins could decrease cell proliferation or promote apoptosis. These include a reduction in different cell signalling systems such as from RAS, RHO, Hedgehog, YAP or TAZ (for review, see Ref. [32]). Furthermore, statins have been shown to reduce N-glycosylation of specific membrane proteins and suppress epithelial–mesenchymal transition (EMT) [33]. Indeed, the reduced levels of cholesterol following statin treatment have also been associated with decreased cancer cell growth [34]. All of these actions of statins, like that of mutant p53 degradation, appear to result from inhibition of HMG-CoA and blockage of the MVA pathway [32].

Consistent with our preclinical studies, several clinical studies have shown that the use of statins was associated with improved outcome in breast cancer patients. Thus following a systematic review of the literature and meta-analysis, Manthravadi et al. [35] identified 10 studies containing 75,684 women with breast, that compared outcome in statin-users versus non-users. Statin use was found to be associated with both improved recurrence-free survival (HR 0.64; 95% CI 0.53–0.79), improved overall survival (HR 0.66; 95% CI 0.44–0.99) and improved cancer-specific survival (HR 0.70; 95% CI 0.46–1.06). Furthermore, in a large population-based study carried out in Denmark, statin use was associated with a reduced risk of breast cancer recurrence in postmenopausal women receiving adjuvant aromatase inhibitors (following multivariate analysis, HR 0.72; 95% CI 0.50–1.04) [36]. Also, in a large randomized phase III double blind clinical trial (BIG 1–98) in which hormone receptor-positive patients were undergoing endocrine treatment, receipt of statins was related to longer disease-free-survival (HR 0.79; 95% CI 0.66–0.95; *p* = 0.01), longer breast cancer-free interval (HR 0.76; 95% CI 0.60–0.97; *p* = 0.02) and longer distant recurrence-free interval (HR 0.74; 95% CI 0.56–0.97; *p* = 0.03) [37].

Of particular relevance to our study was the recent report showing a significant benefit of statins in patients with TNBC but not in those with non-triple-negative disease [38]. Thus, in a large population-based study that included women with stage I-III breast cancer, Nowakowska et al. found that use of statins improved breast cancer-specific survival (HR 0.42; 95% CI 0.20–0.88; *p* = 0.022) and overall survival (HR 0.70; 95% CI 0.50–0.99; *p* = 0.046) in patients with TNBC (*n* = 1534). In contrast, there was no association with breast cancer-specific survival (HR 0.99; 95% CI 0.71–1.39; *p* = 0.97) or overall survival (HR 1.04; 95% CI 0.92–1.17; *p* = 0.55) in those without TN disease (*n* = 15,979). Since cell lines containing mutant p53 appear to be more sensitive to statins than p53 wild-type cells [20] theoretically, the benefit of statins in the tripe-negative cohort relative to the non-TN patients could be a least partly due to the considerable greater prevalence of p53 mutations in the former subtype of breast cancer [5]. In addition to breast cancer, use of statins has also been associated with improved outcome in several other types of malignancy, although contradictory data have also been published including contradictory results in breast cancer (for review, see ref. [39]).

Despite the multiplicity of preclinical and epidemiological studies linking statin use with anti-cancer activity across different types of cancer, there are little data from randomized clinical trials that treatment with these drugs improves outcome for patients with cancer. Statins however, are one of the most commonly prescribed classes of drug worldwide, with well-established safety and dosage profiles [40]. Their limited toxicity, low cost, and ease of use make them an ideal choice for repurposing as anti-cancer drugs.

In conclusion, our data described in this article may be informative with respect to the design of clinical trials involving statins for the treatment of breast cancer. We show that the anti-proliferative effect of simvastatin is enhanced by combination with docetaxel or doxorubicin. Furthermore, our preliminary finding when combined with that of others [20] suggests that statins are more potent cell-growth inhibitors in mutant than in wild-type p53 cells, implying that the mutational status of p53 might be a predictive biomarker for statin sensitivity. Thus, the mutational status of p53 should be investigated in any clinical trial using statins. Finally, while this work was in progress, 2 clinical trials investigating statins in the neoadjuvant treatment of TNBC began recruiting patients (ClinicalTrials.gov Identifier: NCT03358017 and NCT03872388).

## References

[1] Loibl S (2021). Breast cancer. Lancet.

[2] Miglietta F (2022). Major advancements in metastatic breast cancer treatment: when expanding options means prolonging survival. ESMO Open.

[3] Bianchini G (2022). Treatment landscape of triple-negative breast cancer—expanded options, evolving needs. Nat Rev Clin Oncol.

[4] Angus L (2019). The genomic landscape of metastatic breast cancer highlights changes in mutation and signature frequencies. Nat Genet.

[5] The Cancer Genome Atlas Network (2012). Comprehensive molecular portraits of human breast tumours. Nature.

[6] Dang CV (2017). Drugging the ‘undruggable’ cancer targets. Nat Rev Cancer.

[7] Duffy MJ, Crown J (2021). Drugging “undruggable” genes for cancer treatment: are we making progress?. Int J Cancer.

[8] Duffy MJ (2020). Targeting p53 for the treatment of cancer. Semin Cancer Biol.

[9] Miyata Y, Nakamoto H, Neckers L (2013). The therapeutic target Hsp90 and cancer hallmarks. Curr Pharm Des.

[10] Muller PAJ, Vousden KH (2014). Mutant p53 in cancer: new functions and therapeutic opportunities. Cancer Cell.

[11] D'Orazi G, Cirone M (2019). Mutant p53 and cellular stress pathways: a criminal alliance that promotes cancer progression. Cancer (Basel).

[12] Mahalingam D (2009). Targeting HSP90 for cancer therapy. Br J Cancer.

[13] Ramalingam S (2015). A randomized phase II study of ganetespib, a heat shock protein 90 inhibitor, in combination with docetaxel in second-line therapy of advanced non-small cell lung cancer (GALAXY-1). Ann Oncol.

[14] Goyal L, Chaudhary SP (2020). A phase 2 clinical trial of the heat shock protein 90 (HSP 90) inhibitor ganetespib in patients with refractory advanced esophagogastric cancer. Invest New Drugs.

[15] Parrales A (2016). DNAJA1 controls the fate of misfolded mutant p53 through the mevalonate pathway. Nat Cell Biol.

[16] Ingallina E (2018). Mechanical cues control mutant p53 stability through a mevalonate-RhoA axis. Nat Cell Biol.

[17] Synnott NC (2017). Mutant p53: a novel target for the treatment of patients with triple-negative breast cancer?. Int J Cancer.

[18] Chou T-C (2006). Theoretical basis, experimental design, and computerized simulation of synergism and antagonism in drug combination studies. Pharmacol Rev.

[19] Koboldt DC (2012). Comprehensive molecular portraits of human breast tumours. Nature.

[20] Chou C-W (2019). Therapeutic effects of statins against lung adenocarcinoma via p53 mutant-mediated apoptosis. Sci Rep.

[21] Iannelli F (2018). Targeting mevalonate pathway in cancer treatment: repurposing of statins. Recent Pat Anticancer Drug Discov.

[22] Eustace AJ (2019). 1942P—preclinical evaluation targeting both IGF1R and IR in triple negative breast cancer. Ann Oncol.

[23] Rigiracciolo DC (2020). IGF-1/IGF-1R/FAK/YAP transduction signaling prompts growth effects in triple-negative breast cancer (TNBC) cells. Cells.

[24] Sitar T (2006). Structural basis for the inhibition of insulin-like growth factors by insulin-like growth factor-binding proteins. Proc Natl Acad Sci USA.

[25] Bergen K, Brismar K, Tehrani S (2016). High-dose atorvastatin is associated with lower IGF-1 levels in patients with type 1 diabetes. Growth Hormon IGF Res.

[26] Narayanan RP (2013). Atorvastatin administration is associated with dose-related changes in IGF bioavailability. Eur J Endocrinol.

[27] Jang HJ (2016). Statin induces apoptosis of human colon cancer cells and downregulation of insulin-like growth factor 1 receptor via proapoptotic ERK activation. Oncol Lett.

[28] Lee J (2016). Simvastatin induces apoptosis and suppresses insulin-like growth factor 1 receptor in bile duct cancer cells. Gut Liver.

[29] Davison Z (2011). Insulin-like growth factor-dependent proliferation and survival of triple-negative breast cancer cells: implications for therapy. Neoplasia.

[30] Ryan AJ (2009). Expression of a protease-resistant insulin-like growth factor-binding protein-4 inhibits tumour growth in a murine model of breast cancer. Br J Cancer.

[31] Smith YE (2018). Recombinant PAPP-A resistant insulin-like growth factor binding protein 4 (dBP4) inhibits angiogenesis and metastasis in a murine model of breast cancer. BMC Cancer.

[32] Mullen PJ (2016). The interplay between cell signalling and the mevalonate pathway in cancer. Nat Rev Cancer.

[33] Yu R, Longo J (2021). Mevalonate pathway inhibition slows breast cancer metastasis via reduced N-glycosylation abundance and branching. Cancer Res.

[34] Trotta F (2020). Statins reduce intratumor cholesterol affecting adrenocortical cancer growth. Mol Cancer Ther.

[35] Manthravadi S, Shrestha A, Madhusudhana S (2016). Impact of statin use on cancer recurrence and mortality in breast cancer: a systematic review and meta-analysis. Int J Cancer.

[36] Harborg S (2020). Statin use and breast cancer recurrence in postmenopausal women treated with adjuvant aromatase inhibitors: a Danish population-based cohort study. Breast Cancer Res Treat.

[37] Borgquist S (2017). Cholesterol, cholesterol-lowering medication use, and breast cancer outcome in the BIG 1–98 study. J Clin Oncol.

[38] Nowakowska MK (2021). Association of statin use with clinical outcomes in patients with triple-negative breast cancer. Cancer.

[39] Ahmadi M (2020). Pleiotropic effects of statins: a focus on cancer. Biochim Biophys Acta Mol Basis Dis.

[40] Ward NC, Watts GF, Eckel RH (2019). Statin toxicity. Circ Res.

